# Body-kun/body-chan style model figures for artists in forensic visualization applications

**DOI:** 10.1007/s00414-021-02760-3

**Published:** 2022-02-14

**Authors:** Stefan Potente, Sara Heinbuch, Frank Ramsthaler, Peter Schmidt

**Affiliations:** Department of Legal Medicine, University of Saarland Medical School, Kirrberger Straße, Gebäude 49.1, 66421 Homburg/Saar, Germany

**Keywords:** Body position, Visualization, Model, Dummy, Body-kun, Body-chan

## Abstract

Posture and body position are often in the focus of forensic medicine. Visualization for the purposes of documentation, teaching, scientific presentation or expert opinion in court is often desired. Plenty of possible tools to support visualization are available. However, there is a significant gap between quick drawings and more complex techniques. Body-chan (female) and body-kun (male) artist’s model figurines (genericized trademark) may provide a useful means to fill this gap. These models, about 12–15 cm in height, are multi-articulated humanoids of realistic proportions, intended to serve as models for manga (japanese comic) drawing. Plenty of different models are available in different quality which usually are equipped with interchangeable hand and feet attachments, a frame for ‘levitating’ positions as well as assorted objects to scale. These inexpensive models may be positioned quickly and intuitively. Photodocumentation from various angles can be performed using a mobile phone camera. Images may be further improved applying digital image manipulation software. Taken together, the process is quick and intuitive and the level of achievable complexity is sufficient for many forensic applications.

## Introduction: posture, body position and forensic medicine

Understanding the interaction of the human body with its environment, both with other humans and objects, is crucial in forensic medicine. This process involves body positions such as typical and atypical hanging positions, floating positions in water, restraint positions such as ‘hog-tie’ position or ‘incaprettamento’ [1] and different forms of positional asphyxia, with body position itself significantly contributing to the occurrence of death. Stabbing, punching, kicking and strangulation exhibit many variations (such as ‘burking’ [2]) which demand more detailed clarification. A powerful visualization may substantially facilitate documentation, teaching and training as well as scientific and judicial presentations. As far as dead body positions are concerned photos from the scene may naturally often be used (see, for example, [3–5]). However, space constraints and other aspects of the situation may not allow for taking ‘fully descriptive’ photographs (see, for example, [6]). Generally and put simply, there are several visualization tools for posture and body position available:

- **Simple drawings**, such as ‘stick figures,’ may be sufficient to illustrate different stabbing techniques [7], a person jumping and landing [8] or a person dragged underneath a car [9]. However, drawings are low in detail and leave room for aesthetical improvement.
- **Traditional wooden artist’s figures** resemble correct body proportions but are otherwise extremely limited in articulation and overall complexity. They are mentioned here merely for completeness.
- **Complex drawings and illustrations** require considerably more time and skill to produce. Consistency and perspective may be particularly challenging. The combination of photographic demonstrations with complex drawings may add significant value to the demonstration, for example, as shown in the chapter on neck holds in [10].
- **Photographic demonstration** is put to good use in many forensically relevant visualizations (for example, [11–13]). It takes planning, staff and technical know-how. Furthermore, many relevant positions are not suitable for this technique, for example, depictions of dynamic impacts, dangerous actions (for example, squatting on one’s chest) or depictions of sexual violence. On rare occasion, some aspects of the body position may be demonstrated and photodocumented during autopsy (see Fig. 5).
- **Computer-assisted rendering** can produce good visual results for all imaginable body positions and angles (see, for example, [14]). Programs such as Poser®; [15] (for example, here, [16] and Fig. 5) have made this process more accessible. There may be a considerable learning curve for the user, especially when the program is used only infrequently. The realistic posing of a high-resolution humanoid model must be distinguished from biomechanical computer modelling, such as the accident reconstruction software PC-crash [17] (used, for example, in [18, 19]), which often reduces the complexity of humanoid models as part of the simulation.

Since there is a considerable gap between quick yet limited simple drawings and more detailed yet much more involved methods, we intend to present modern artist’s model figurines as a visualization method which is both quick and sufficiently detailed for many forensic purposes.

## Method: body-kun and body-chan style artist’s figurines

For some time now, a new generation of articulated, realistically proportioned artist’s models is available. The best distinction between these and the traditional wooden artist’s figurines that we found was the search terms ‘body-kun’ [20] or ‘body-chan’ as a genericized trademark or proprietary eponym for such objects.^1^ As the names suggest (‘kun’ ( ) being the japanese honorific for male children and ‘chan’ ( ) for female children), these models are intended for manga drawing.^2^ They are not toys.

**Fig. 1:**
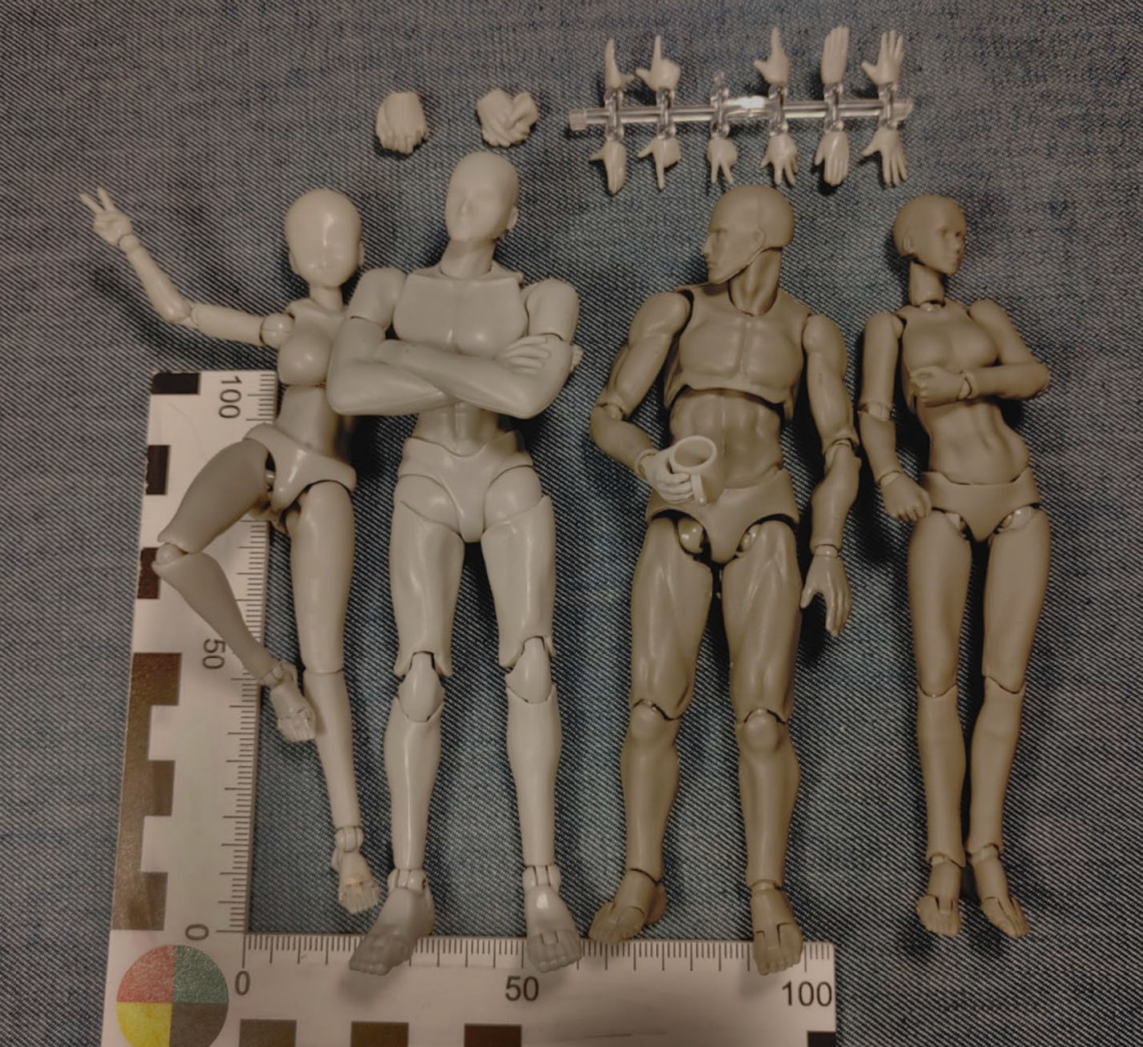
Two different male/female sets of body-kun/chan style figurines. Note the shiny surface and lower overall detail on left pair (brand ‘S.H.Figuarts’) compared to the darker figurines on the right (brand ‘lzn Body Chan & Kun’). Second figure from the left features ‘folded arms’ attachment. Third figure from the left holds a scaled coffee mug included in the set. Note different hand gestures, with additional replacements shown on top

In contrast to traditional wooden models, these models are usually made of different kinds of plastic and well articulated, both in number and in degrees of freedom (see Fig. 1). Joints are pressure fit and thus allow for changing of parts. Levels of definition in facial expressions and muscle shapes vary. Models are usually marketed in packages containing both a male and a female model as well as interchangeable hands and feet (including ‘folded arms’ and ‘folded hands,’ see Figs. 1 and 4) furthermore some clear plastic frame for ‘levitating’ dynamic positions (jumping, kicking, see Fig. 3). Some brands include additional objects such as coffee mugs, laptops or weapons. Price is usually below 30 USD for a male-female set. The height of the figures is usually around 12 to 15 cm.

## Results

The following items/constellations were identified as useful applications.

**Fig. 2:**
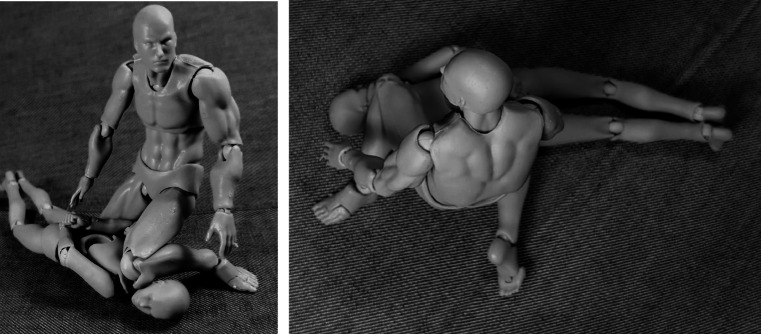
Depiction of ‘knee on neck’ position, circumventing copyright issues (left) and allowing a new perspective (right)

**Fig. 3:**
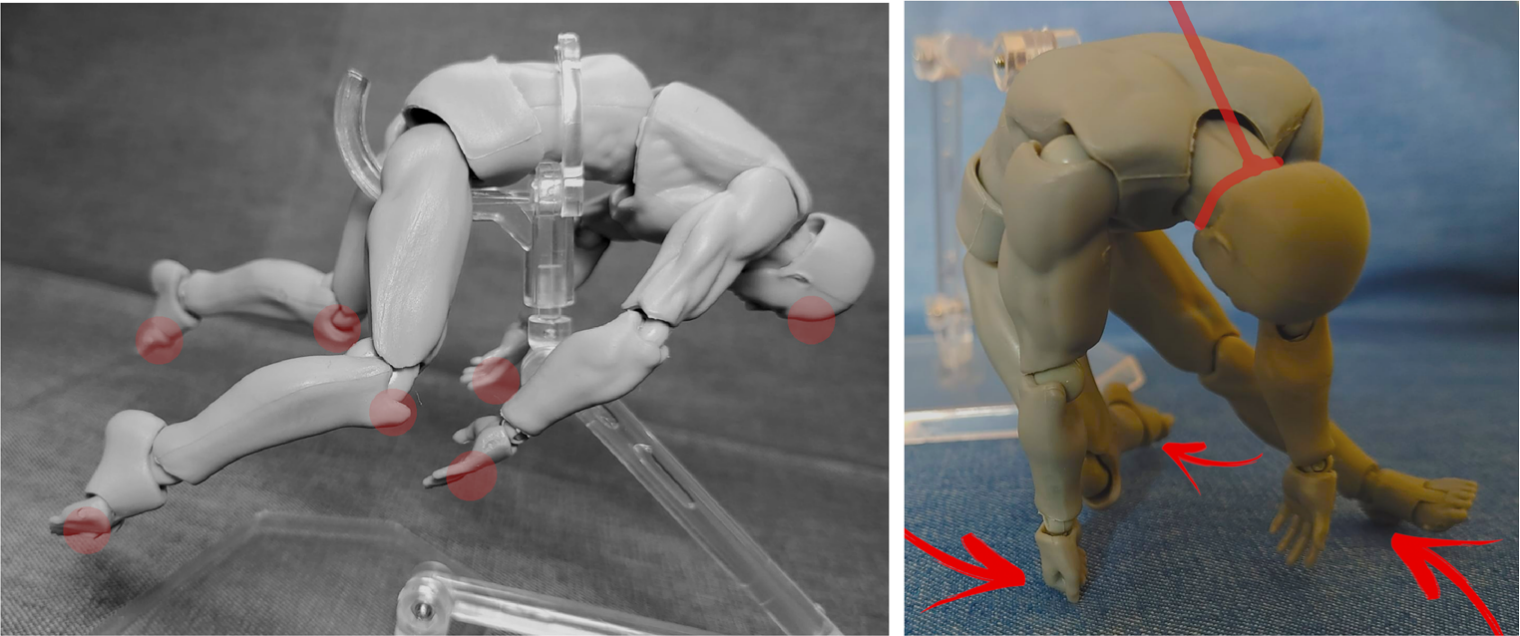
Use of adjustable stand and image processing (Gimp 2.10). Left: ‘Floating in water’ body position with grayscaling and color marking of common floating defects. Right: Position of suspension with points of contact with the ground marked (arrows) and ligature position drawn

**Fig. 4:**
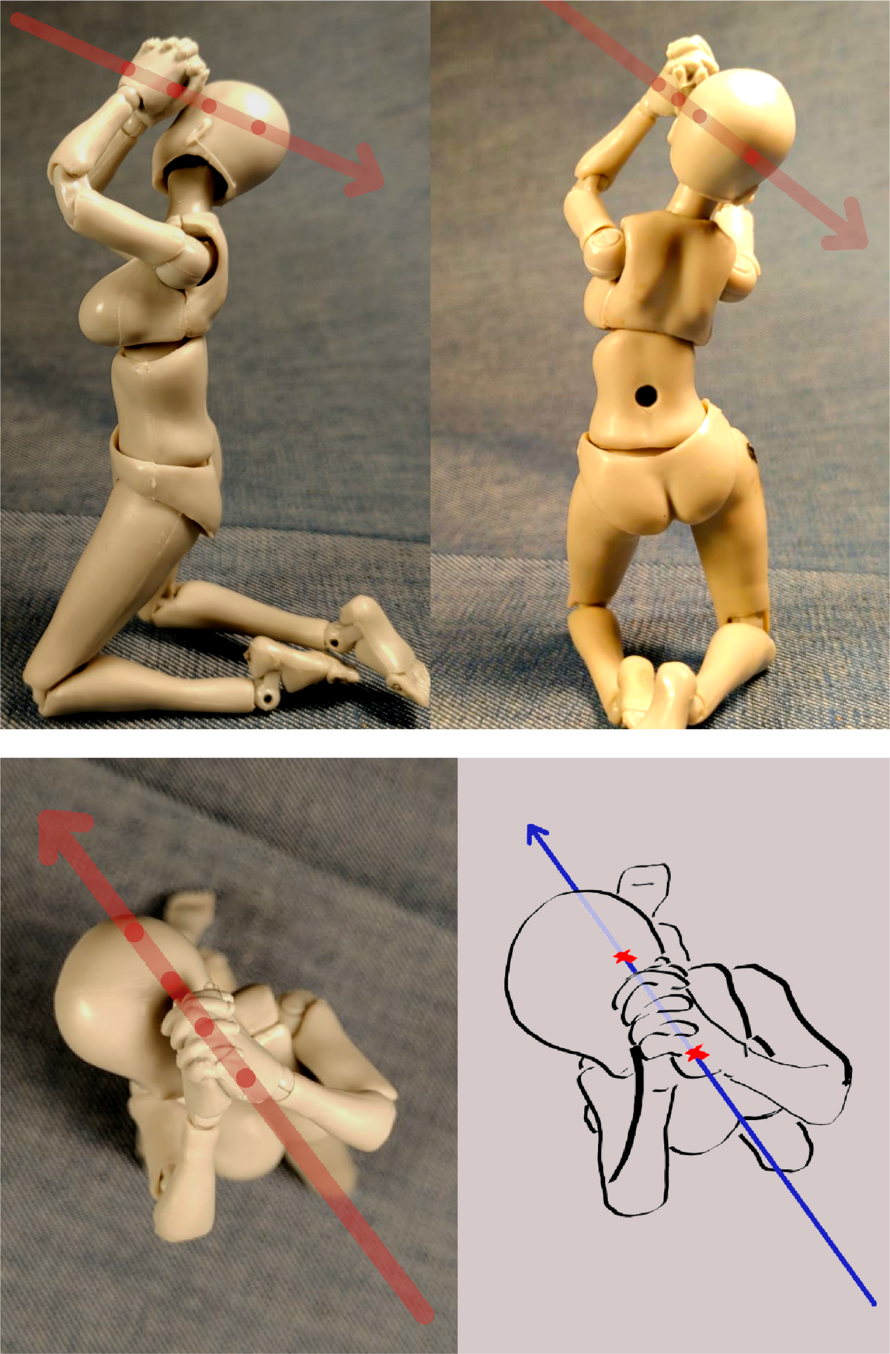
Top row and lower left: Visualization of GSW tract and body position, using the ‘folded hands’ attachment. Hole in the back (top right) is for attaching a clear plastic rod for upright positioning. Saturation, contrast, arrow inserts: Gimp 2.10. Lower right: Drawing for clarification using superimposition: Inkscape 1.0.2

**Fig. 5:**
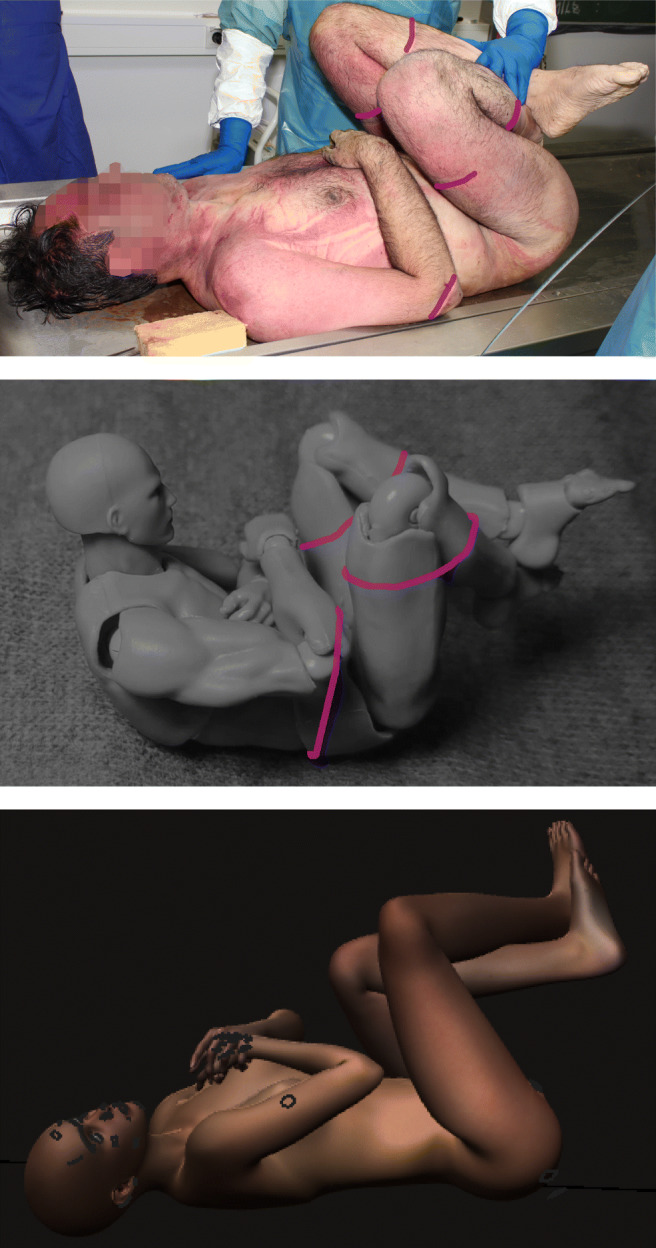
**Top:** Reconstruction of body position during autopsy in a case of a man recovered from a shallow grave who had been transported in a box-shaped textile bag. Added pink lines mark gaps in lividity, imprints and dry markings, indicative of binding or contact with seams of bag. **Middle:** Visualization using body-kun figurine. Note position of arms and head and also limited flexing in hip joints. **Bottom:** Visualization using Poser Pro®;, version 11.0

- Interaction of one or several models, with consistant documentation from different angles.
- Basis for further processing, either as drawing into the picture as well as drawing over the picture using programs such as Inkscape [21], respectively other free or commercial image processing software such as Gimp [22] (see, for example, Figs. 4 and 5).
- Depiction of floating, hanging (see Fig. 3) or dynamic impact positions using the provided stand.
- Reverse engineering from original photographs to circumvent copyright issues and gain additional perspectives (see Fig. 2)

The informative value is exemplarily shown in Figs. 2 and 3. Use of the models was intuitive yet potentially finicky. Documentation was as simple as taking a picture with a mobile phone.^3^ Use of additional photography equipment may improve the image quality but is not imperative. Equally, the images may be processed further using image processing software for improving brightness and contrast, color saturation, grayscaling, cropping, coloration and drawing in lines, arrows, writing and such. The process of positioning the model(s) and photo documentation from different angles, such as in Figs. 2 and 4, was only a matter of minutes. We found no major drawbacks compared to computer visualization of *relatively simple* body positions for demonstration purposes when compared to computer rendering (Fig. 5).

## Discussion

On the one hand the use of body-kun style figurines was confirmed to be cost-effective, time-efficient, intuitive (‘un-unlearnable’) and minimalistic to set up. On the other hand some products in online searches were assessed to be unsuitable for use in the forensic context (‘children with breasts’ and ‘super hero physique’). Some minor practical handling problems were encountered, where in photodocumentation lighting may be problematic for shiny plastic surfaces of some brands. Photodocumentation of details may produce shallow depth-of-field issues. Pressure fit plastic joints are prone to breaking with extended use and small parts may be lost easily. Not all joints allow for extreme flexing and extension, which we found most notable in the hip joints.

To sum up, despite some minor resolvable difficulties of practical handling, these figurines were proven to be useful tools which may close the gap between a simple drawing and more involved techniques such as computer rendering for the demonstration of body positions.

## Footnotes

1 Many companies make their products available through these search terms while often selling knock-offs and copies which make it difficult to identify original products of good quality.

2 Manga ( ): Japanese comic books/graphic novels.

3 All photographic images in this publication (except top image in Fig. 5) were taken with the author’s phone (LG G80 ThinQ Dual Screen).
